# Clinical evaluation of hypercoagulability in advanced malignant tumors using thromboelastography and conventional coagulation tests

**DOI:** 10.1097/MD.0000000000041465

**Published:** 2025-02-07

**Authors:** Jinzhu Yang, Lei Shen

**Affiliations:** a Department of Oncology, The Third Clinical College of Anhui Medical University (The Third People’s Hospital of Hefei), Anhui, China.

**Keywords:** conventional coagulation tests, hypercoagulability, malignant tumors, thromboelastography

## Abstract

This study compares the diagnostic value of conventional coagulation tests (CCTs) and thromboelastography (TEG) for high blood coagulation states in advanced malignant tumors and to explore the diagnostic efficacy of their combination. In this study, 120 patients with advanced malignancy were divided into hypercoagulable state (n = 62) and non-hypercoagulable state (n = 58) groups. Traditional coagulation tests or CCTs (including prothrombin time, activated partial thromboplastin time, international normalized ratio, fibrinogen, D-dimer, and platelet count) were conducted. Simultaneously, TEG parameters, such as kinetic time, reaction time, clotting angle, maximum amplitude, and coagulation index, were assessed. Baseline demographic and pathological data were also collected. The role of conventional coagulation indicators, TEG indicators, and their combination in diagnosing high blood coagulation states was explored. The diagnostic efficiency was evaluated by constructing curves and calculating the area under the curve (AUC). Among 120 patients with advanced malignancy, 51.67% (62/120) had a hypercoagulable state. Among CCTs, activated partial thromboplastin time, D-dimer, and platelet count were associated with a hypercoagulable state, whereas no significant differences were found in other indicators. Among TEG parameters, maximum amplitude, reaction time, and clotting angle were associated with a hypercoagulable state, whereas kinetic time and coagulation index were not significantly different. The combined use of CCTs and TEG parameters was more effective in diagnosing hypercoagulable states than either test alone. The AUC values for the diagnostic efficacy of the CCTs, TEG, and TEG combined with CCTs for the diagnosis of hypercoagulable transitions in blood were 0.825, 0.744, and 0.947, respectively, with the highest AUC value in the combined test group. This study indicates that TEG parameters were highly correlated with hypercoagulability in patients with malignant tumors. The combined use of CCTs and TEG parameters is more effective for diagnosing hypercoagulability. These results can guide the clinical management and treatment of patients with malignant tumors.

## 1. Introduction

Hypercoagulability, a condition characterized by an increased propensity for blood clot formation, is commonly observed in patients with advanced malignant tumors.^[1]^ This is of particular concern, as hypercoagulability is associated with poor prognosis, increasing the risk of complications such as venous thromboembolism.^[2]^ Thus, it is essential to identify effective diagnostic methods for hypercoagulability to improve the clinical management and treatment outcomes in patients with late-stage malignancies.^[3]^

In this study, we aimed to compare the diagnostic value of conventional coagulation tests (CCTs)^[4]^ such as prothrombin time, activated partial thromboplastin time (APTT), international normalized ratio, fibrinogen (FIB), D-dimer (D-D), and platelet count (PLT). Thromboelastography (TEG) indicators^[5]^ include kinetic time (K), reaction time ®, clotting angle (Angle), maximum amplitude (MA), and coagulation index (CI). TEG is a more comprehensive method for assessing coagulation function. Furthermore, we explored the diagnostic efficacy of combining CCTs and TEG parameters for detecting hypercoagulability.

The results of our investigation provide valuable insights into the clinical evaluation of hypercoagulability in patients with advanced malignant tumors, shedding light on the diagnostic potential of TEG and the benefits of combining it with CCTs.

## 2. Methods

### 2.1. Study population and design

This study focused on patients diagnosed with advanced malignant tumors who were admitted to the Department of Oncology at the prestigious Hefei Third People’s Hospital from January 2019 to January 2023. A total of 120 patients (75 males and 45 females) with a mean age of (65.50 ± 8.80) years and varying tumor types including lung, gastric, colorectal, and liver cancer were included in this study. Based on the presence or absence of embolism, patients were further classified into the blood hypercoagulable state group (n = 62) and non-hypercoagulable state group (n = 58). Ethical clearance for this study was granted by the Medical Ethics Committee of the hospital (Reference Number: 2023LLWL016), and consent was obtained from all participating patients. All relevant data are within the paper and its supporting information files.

### 2.2. Criteria for selection

The inclusion criteria were as follows: clear pathological data for tumor diagnosis and confirmation of advanced malignant tumors. Within 3 weeks, they had not received radiotherapy, chemotherapy, or anticoagulant drug treatments, nor had any major surgical history. The exclusion criteria included potential confounding factors affecting coagulation function, such as hematologic or autoimmune diseases, and severe liver or kidney function impairment.

### 2.3. Detection methods

The testing instrument used was the UD-T8000 thromboelastograph (Shenzhen Youdi Biotechnology Co., Ltd.), which employs the whole blood compound calcium method for assessment. To conduct the test, 38 g/L sodium citrate anticoagulated blood (blood-to-anticoagulant ratio of 9:1) was collected, taking 0.6 mL and placing it into a plastic or silicon-coated test tube. Subsequently, a 12.9 g/L CaCl_2_ solution of 0.4 mL is added and mixed. The stopwatch was started immediately. Finally, 0.36 mL of the mixture is measured in a blood cup.

An additional patient’s morning fasting venous blood sample of approximately 2 mL (as per the blood collection tube scale) was collected and anticoagulated with EDTA-K2. The sample was thoroughly mixed and inverted before being tested on a BC-5180 fully automated hematocrit analyzer after a half-hour interval. Quality control measures were implemented using Myriad Corporation’s internal quality control. The manufacturer’s reagents were used to detect PLT levels.

### 2.4. Definition of hypercoagulable (high condensation) and non-hypercoagulable states

Patients were considered hypercoagulable if they exhibited shortened R, elevated MA, and/or increased CI on TEG, accompanied by abnormally high conventional coagulation parameters (e.g., D-D, FIB). Those not meeting these criteria were classified as “non-hypercoagulable.” This definition aligns with published recommendations.^[4]^

### 2.5. Sample size estimation

We employed a single-proportion formula to determine the required sample size for estimating the prevalence of hypercoagulability in patients with advanced malignancies. The single-proportion formula is: n = Z^2^_α/2_ × p(1 − p)/d^2^. According to previous studies, the prevalence of hypercoagulability can reach 73.8%.^[6]^ Using this value (*P* = .738) and setting α = 0.05 (two-sided) and an allowable error of d = 0.08, the formula yielded n ≈ 116. Considering possible dropouts and loss to follow-up, we ultimately enrolled 120 patients to ensure adequate sample size and robustness of our findings.

### 2.6. Statistical analysis

The IBM SPSS software (version 26.0) was used for data analysis. Count data were described statistically using composition ratios or rates, and a comparative analysis was conducted using the *χ^2^* test. D-D and CI were described by [M(P25, P75)] and statistically analyzed using the Wilcoxon rank sum test. Multifactor analysis was performed using unconditional dichotomous logistic regression models and the odds ratios and 95% confidence intervals were calculated. Diagnostic efficacy predictions were evaluated using subject operating characteristic curves, and the area under the curve (AUC) was determined. Statistical significance was set at *P* < .05.

## 3. Results

### 3.1. Baseline data

The general conditions of both groups were compared separately, including age, sex, diabetes, hyperlipidemia, smoking, alcohol abuse, and tumor type. The results indicated no statistically significant differences between the groups (all *P* > .05). Table 1 provides a detailed overview of these results.

**Table 1:** Baseline information of patients in both groups [(x̅±s), cases (%)].

Parameters	Hypercoagulable state group (n = 62)	Non-hypercoagulable state group (n = 58)	*t/χ2*	*P*
Age	66.26 ± 8.84	64.29 ± 8.75	-1.223	.224
Gender (male/female)	39 (62.90)/23 (37.10)	36 (62.07)/22 (37.93)	0.009	.925
Diabetes	17 (27.42)	15 (25.86)	0.037	.847
Hyperlipidemia	13 (20.97)	11 (18.97)	0.075	.784
Smoking	33 (53.23)	30 (51.72)	0.027	.869
Alcoholism	21 (33.87)	18 (31.03)	0.110	.740
Tumor type			1.867	.601
Lung cancer	24 (38.71)	25 (43.10)		
Stomach cancer	15 (24.19)	17 (29.31)		
Colorectal cancer	10 (16.13)	9 (15.52)		
Liver cancer	13 (20.97)	7 (12.07)		

*P*: *t* test was used for age and chi-square test was used for other parameters.

### 3.2. Differences in CCTs and TEG between groups

CCTs and TEG results were compared between the 2 groups. The findings indicated significant differences between the MA, R, and Angel groups in the TEG parameters (all *P *< .05). Additionally, statistically significant differences were observed between the APTT, D-D, and PLT groups in CCTs parameters (all *P* < .05). For further details, please refer to Table 2.

**Table 2:** Results of TEG and CCTs examinations in both groups (x̅±s) [M(P25, P75)].

Parameters	Hypercoagulable state group (n = 62)	Non-hypercoagulable state group (n = 58)	*t/χ^2^/Z*	*P*
CCTs				
PT (S)	12.31 ± 1.60	12.17 ± 1.78	-0.433	.666
APTT (S)	27.32 ± 3.98	31.16 ± 4.38	5.017	<.001
INR	0.93 ± 0.10	0.94 ± 0.10	0.902	.369
FIB (g/L)	3.72 ± 0.59	3.58 ± 0.61	-1.263	.209
D-D (g/L)	3.23 (1.80, 4.18)	0.41 (0.11, 0.78)	-7.613	<.001
PLT (×10^9^/L)	305.79 ± 59.72	246.62 ± 43.02	6.190	<.001
TEG				
K (min)	2.09 ± 0.50	2.01 ± 0.42	0.900	.370
R (min)	8.44 ± 3.06	6.47 ± 1.69	4.331	<.001
Angle (deg)	54.82 ± 9.43	60.78 ± 12.04	3.030	.003
MA (mm)	59.94 ± 5.36	64.69 ± 9.38	-3.436	.001
CI (min)	-0.18 (-1.59, 1.83)	-0.81 (-2.79, 1.26)	-1.541	.123

*P*: Wilcoxon rank sum test was used for D-D and CI and *t* test was used for other parameters.

APTT = activated partial thromboplastin time, CCTs = conventional coagulation tests, CI = coagulation index, D-D = D-dimer, FIB = fibrinogen, K = kinetic time, MA = maximum amplitude, PLT = platelet count, PT = prothrombin time, R = reaction time, TEG = thromboelastography.

### 3.3. Multifactorial analysis

The variables that exhibited statistically significant differences between the aforementioned groups (APTT, D-D, PLT, MA, R, and Angel) were used as independent variables. The presence or absence of blood hypercoagulation (presence = 1, absence = 0) was employed as the dependent variable for the unconditional dichotomous logistic regression analysis. The optimization model was fitted using a stepwise backward method. The findings revealed that APTT, PLT, R, and Angel were independent risk factors associated with the development of a hypercoagulable blood state in patients with malignancy (all *P* < .05). For complete details, please refer to Table 3.

**Table 3:** A multifactorial analysis of factors affecting the hypercoagulable state of blood in patients with advanced malignancies.

Parameter	β	SE	Wald	*P*	OR	95% CI Lower	95% CI Upper
APTT	-0.294	0.094	9.821	.002	0.745	0.620	0.896
D-D	1.325	0.818	2.624	.105	3.764	0.757	18.690
PLT	0.013	0.005	6.760	.009	1.013	1.003	1.023
MA	-0.171	0.098	3.045	.081	0.843	0.696	1.021
R	0.298	0.130	5.228	.022	1.348	1.044	1.740
Angel	-0.031	0.011	7.942	.005	0.969	0.949	0.991

APTT = activated partial thromboplastin time, D-D = D-dimer, MA = maximum amplitude, PLT = platelet count, R = reaction time.

### 3.4. Diagnostic efficacy

**Figure 1.**
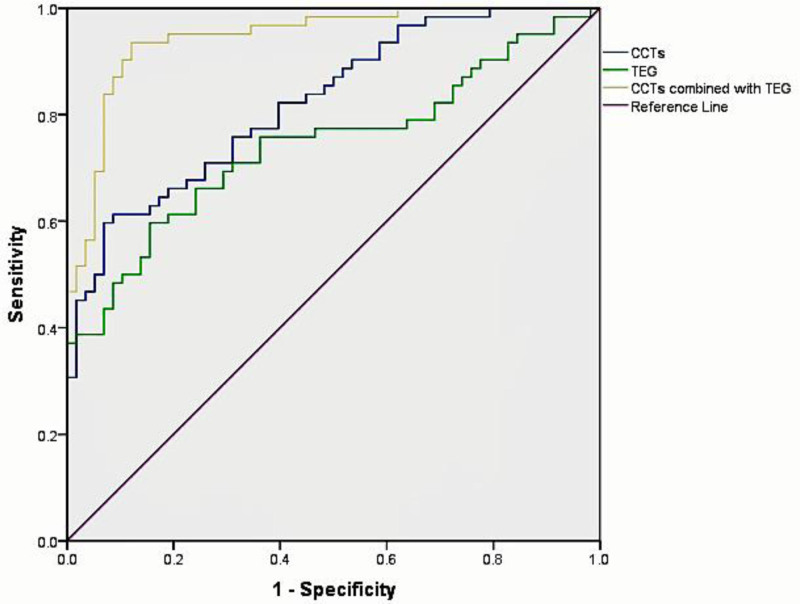
Diagnostic efficacy of 3 diagnostic methods for patients with advanced malignant tumor.

Subject operating characteristic curves were plotted separately for CCTs parameters (APTT, PLT), TEG parameters (R, Angel), and CCTs combined with TEG to predict adverse blood hypercoagulability. The results indicated that CCTs parameters, TEG parameters, and CCTs combined with TEG exhibited good predictive values for the development of blood hypercoagulability in patients with advanced malignancies. The AUC values for CCTs parameters, TEG parameters, and CCTs combined with TEG were 0.825, 0.744, and 0.947, respectively. However, the combination of CCTs and TEG exhibited the highest diagnostic efficacy (Table 4 and Fig. 1).

**Table 4:** Diagnostic efficacy of CCTs, TEGs, and CCTs combined with TEG in the diagnosis of hypercoagulable state of blood in patients with advanced malignancies.

Parameters	AUC	Yoden index	Sensitivity	Specificity
CCTs	0.825	0.528	0.597	0.931
TEG	0.744	0.442	0.597	0.845
CCTs combined with TEG	0.947	0.784	0.887	0.897

AUC = area under curve, CCTs = conventional coagulation tests, TEG = thromboelastography.

## 4. Discussion

Coagulation is a dynamic process that involves a series of enzymatic reactions and coagulation factors.^[7]^ Traditional coagulation tests do not accurately represent the coagulation status of the body and have low predictive value for thromboembolic events. TEG can monitor dynamic shifts in blood coagulation and offer a comprehensive understanding of coagulation status in patients.^[8]^ TEG is extensively employed across numerous fields, including surgery,^[9,10]^ blood transfusion,^[11,12]^ and anticoagulation therapy,^[13,14]^ among others. Key parameters of the TEG assay and their significance: (1) R value: the time required from the initiation of blood sample detection to the initial clot formation. The R value is predominantly influenced by coagulation factors and anticoagulants. (2) K value: the time elapsed from the initiation of clot formation to the attainment of a specific clot strength (MA amplitude of 20 mm). As the function increases, the K value decreases. The K value is primarily affected by the function and level of FIB. (3) Angle: the angle formed between the tangent line and the horizontal line, measured from the point of clot formation to the maximum arc of tracing. This angle represents the rate of fibrin formation. (4) MA value: the maximum amplitude of the tracing, indicative of the clot’s maximum strength. It is dependent on the number and functionality of platelets. The MA value increased with an increase in platelet function. CI value: the comprehensive coagulation status was determined based on R, K, Angle, and MA values. The CI typically falls between −3 and + 3. A CI value below −3 signifies a low coagulation state, whereas a CI value above + 3 indicates a hypercoagulable state.

This study compared the diagnostic value of CCTs and TEG in identifying hypercoagulability in patients with advanced malignant tumors. Hypercoagulability is associated with a poor prognosis, making it crucial to identify effective diagnostic methods. Among CCTs, APTT, D-D, and PLT were found to be significantly associated with hypercoagulable states, which is consistent with a previous prospective study.^[15]^ In comparison, the TEG parameters MA, R, and Angel also showed significant associations with hypercoagulability.^[16]^ However, there were no significant differences in the other indicators between CCTs and TEG. The combined use of CCTs and TEG parameters has been shown to be more effective in diagnosing hypercoagulability in patients with advanced malignant tumors. This study demonstrated that combining these tests enhanced the diagnostic efficacy compared to either test alone. The AUC values for the diagnostic efficacy of the 3 test options (CCTs, TEGs, and CCTs combined with TEGs) were 0.825, 0.744, and 0.947, respectively. Importantly, the highest AUC value was observed in the combined test group, emphasizing the superiority of the combined approach in diagnosing hypercoagulability in patients with malignant tumors. This insight can significantly contribute to clinical management and treatment strategies for these patients.

The importance of diagnosing hypercoagulability in patients with malignant tumors cannot be overstated, as it plays a crucial role in overall clinical management and treatment outcomes.^[17,18]^ Accurate and timely diagnosis of hypercoagulability can lead to better targeted interventions, reducing the risk of complications, such as deep vein thrombosis,^[19]^ pulmonary embolism,^[20]^ or even death. The hypercoagulable state of blood after tumor resection is an area of concern for clinicians.^[21]^ Theoretically, as tumor factors diminish following surgery, blood slowly recovers to a normal coagulation state. However, previous studies have shown otherwise.^[22]^ The hypercoagulable state continues to worsen, which may be linked to several factors. First, surgical trauma causes venous stasis and leads to vascular endothelial damage, which in turn triggers blood coagulation and worsens blood hypercoagulability. Additionally, postoperative patients’ reduced physical activity and slow blood flow contribute to a hypercoagulable state. Moreover, intravenous nutrition via deep vein tubes, usually administered to most patients with advanced tumors, can damage the vascular endothelium and promote blood coagulation. Therefore, clinicians must be vigilant in managing postoperative coagulation to minimize the risk of thrombosis.

This study has the following limitations. First, the study design was prone to bias and confounding factors that may affect the accuracy of the results. Second, the study relied on the accuracy and completeness of the medical records, which may be incomplete or inaccurate. Third, the study may not account for changes in treatment protocols or patient characteristics over time, which may have affected the outcomes. Finally, the study may not be generalizable to other populations or settings, as it only included a specific group of patients with malignancy. Therefore, caution should be exercised when interpreting the results of retrospective studies, and further research is needed to confirm these findings.

## 5. Conclusion

A study on the efficacy of TEG in patients with advanced malignancies revealed that combining TEG with CCTs tests is highly effective in detecting the hypercoagulable state of the blood in such patients. This finding has significant implications for clinical practice as it suggests that the use of these tests can aid in the diagnosis and management of thrombotic events in patients with advanced malignancies. By identifying the hypercoagulable state of the blood, physicians can take appropriate measures to prevent and treat thrombotic events, which can be life-threatening in some cases. Overall, this study underscores the importance of TEG in the management of patients with advanced malignancies.

## Author contributions

**Conceptualization:** Lei Shen.

**Data curation:** Jinzhu Yang, Lei Shen.

**Formal analysis:** Jinzhu Yang, Lei Shen.

**Funding acquisition:** Lei Shen.

**Investigation:** Jinzhu Yang, Lei Shen.

**Methodology:** Jinzhu Yang, Lei Shen.

**Resources:** Jinzhu Yang.

**Writing – original draft:** Jinzhu Yang, Lei Shen.

**Writing – review & editing:** Lei Shen.
